# Examining Community Preferences for Adaption and Dissemination of an Efficacious Brief Intervention on Alcohol Use and Sexual Behavior

**DOI:** 10.1177/15248399241291866

**Published:** 2024-11-18

**Authors:** Stacey B. Griner, Idara Akpan, Kaeli C. Johnson, Grace Maynard, Sarah A. Alkhatib, Nathaniel J. Webb, Annalynn Galvin, Nolan Kline, Dana M. Litt, Melissa A. Lewis, Erika L. Thompson

**Affiliations:** 1 The University of North Texas Health Science Center, Fort Worth, TX, USA; 2 UT Health Houston, Houston, TX, USA; 3 University of Central Florida, Orlando, FL, USA; 4 The University of Texas Health Science Center at San Antonio, San Antonio, TX, USA

**Keywords:** alcohol use, sexual behavior, interventions, health communication, dissemination channels, young adults, community stakeholders

## Abstract

Compared to other age groups, 18- to 25-year olds (young adults) are more likely to engage in heavy alcohol use and inconsistent contraceptive use, increasing their susceptibility to sexually transmitted infections (STIs) and unintended pregnancy. The Studying Alcohol and Related Risks (STARR) intervention was efficacious in reducing young adult alcohol-related risky sexual behavior, including reducing the number of casual sexual partners and alcohol use prior to sex. We conducted a qualitative study to guide the adaptation of the STARR intervention to include additional content on contraceptive use and prepare for dissemination of the intervention to a community audience. We conducted 10 focus groups with young adults (n = 16) and semistructured interviews with local community stakeholders (n = 12) to examine: (a) intervention characteristics, such as compatibility, adaptability, and design and packaging and (b) dissemination and communication channels. Focus groups and interviews were audio-recorded, transcribed, and thematically analyzed. Participants found the proposed intervention acceptable and highlighted the need to promote STI prevention among young adults. Participants viewed text-based interventions as accessible and effective. Key considerations included developing personalized messages from credible sources, using gender-inclusive language, and sending messages at strategic timepoints. Social media (Instagram, Twitter, Snapchat, TikTok), events, and campus resources were described as avenues to create awareness and disseminate information about the proposed intervention. Findings demonstrate the need for innovative and tailored young adult health programs that incorporate multilevel dissemination strategies. This study highlights the need for implementation activities that will improve the adoption and dissemination of evidence-based programs, particularly among young adults.

Sexual health practices such as sex without contraception or a condom can lead to elevated risk of sexually transmitted infections (STIs) and unintended pregnancy (Centers for Disease Control and Prevention [CDC], 2024). Young adults are particularly susceptible to the adverse consequences of these behaviors, as half of new STIs in 2022 were among 15- to 24-year olds (CDC, 2024), and women ages 18 to 24 report high rates of unintended pregnancies (Guttmacher Institute, 2019). Among the leading risk factors for adverse sexual health and pregnancy outcomes among young adults is alcohol misuse. Alcohol consumption may lead to sex without contraception, condomless sex, or reduce an individual’s ability to consider the long-term consequences of these behaviors (Brown et al., 2016). The strong association between inconsistent condom use, multiple sex partners, and alcohol use among young adults has led to a call for prevention efforts across multiple social settings, including at home, in schools, and in communities (Cho & Yang, 2023).

Existing interventions for reducing sex without contraception or condomless sex that co-occur with alcohol use have not been generalizable to community-based samples due to their focus on college students, despite not all young adults attending college (Ahankari et al., 2019; Kilwein et al., 2017). The STudying Alcohol and Related Risks (STARR) intervention was developed to address this issue by providing a brief web-based intervention to a national young adult sample that had not used a condom during sex alongside alcohol use in the past month, among other inclusion criteria (Lewis et al., 2019). The STARR intervention used personalized feedback delivered via the web for participants and focused on alcohol expectancies, normative comparisons, and protective behavioral strategies to reduce alcohol-related sexual health outcomes (Lewis et al., 2019). The feedback component of the intervention was developed in alignment with other efficacious interventions using personalized feedback related to sexual health and alcohol use (Li et al., 2022). Most participants engaged with the personalized feedback component of the intervention once for 5 to 7 minutes, and at 1 month following the intervention, there was reduction in alcohol-related sexual consequences (using a 41-item scale; Fairlie et al., 2021), the number of times drinking before sex, and the number of casual sexual partners (Lewis et al., 2019). More recent assessments demonstrate that many participants completed the intervention in one session (~53%–54%) and returned to the intervention more than once (43%). Moreover, the average duration of viewing the intervention was 3.5 minutes (Li et al., 2022).

Additional strategies are needed to extend the impact of the STARR intervention to address unintended pregnancies by including contraceptive use and expanding the intervention’s dissemination to the broader community. The goals of this pilot study include identifying areas for adaptation and ultimately developing dissemination plans for the two groups of end users—community partners/organizations and young adults—following intervention adaptation and evaluation. The STARR intervention was efficacious at improving alcohol-related sexual consequences, but focused on condom use to reduce STI risk and did not include information on the prevention of unintended pregnancies (Lewis et al., 2019). Expanding components of the intervention that include condom and contraceptive co-use will increase the impact of the intervention on alcohol use, STI prevention behaviors, and unintended pregnancy, and adaptation of evidence-based sexual health interventions has been shown effective in past work (Javidi et al., 2021; Kachingwe et al., 2023). Based on the previous work related to this intervention, we anticipate this program would be utilized by and recommended to sexually active young adults aged 18 to 25 who report heavy episodic drinking in the past month (Lewis et al., 2019). While the STARR intervention showed evidence of efficacy at reducing alcohol-related sexual consequences (Lewis et al., 2019), the intervention has yet to be implemented widely in a community setting, specifically with local community organizations who may use or recommend this intervention to their clients. Research is needed relating to the dissemination and implementation of the STARR intervention and how to most appropriately integrate content meant to reduce unintended pregnancies during alcohol-related sexual experiences and prepare for intervention dissemination.

The purpose of this pilot study was to conduct focus groups with young adults and interviews with community stakeholders to gather preliminary data that will inform the adaptation of an efficacious brief intervention (STARR intervention) on alcohol use and sexual behavior (including contraceptive use and STI prevention behaviors). We elicited feedback on the intervention, including: (a) intervention characteristics, such as compatibility, adaptability, and design and packaging; and (b) dissemination and communication channels and strategies. The information gained here can be used to improve the dissemination and implementation of the adapted intervention into community settings and identify strategies to promote intervention adoption among both stakeholders and young adults.

## Method

### Conceptual Framework

The study was guided by the Diffusion of Innovation (DOI) model to inform intervention adaptation, dissemination, and future adoption. DOI focuses on how innovations, such as ideas, interventions, and behaviors, passively spread through communities and are ultimately adopted into society (Rogers, 2003). The model posits that *adopters* (individuals who use the intervention) have *prior conditions* and *individual characteristics* that can influence their *decision to adopt* the intervention (Rogers, 2003). The model suggests that individuals can be persuaded through *dissemination and communication channels* (sources of information) to adopt the intervention if the *intervention characteristics (relative advantage, compatibility, and complexity)* meet their needs (Rogers, 2003). Data using this theory were collected using two approaches: focus groups with young adults (called focus group participants from here on) and interviews with community stakeholders, and data were synthesized during analysis (see Table 1 for eligibility criteria for focus group participants [young adults] and community stakeholders). For this pilot study, we applied DOI viewing focus group participants (young adults) as the intervention’s adopters. For community stakeholders, we also view them as adopters of the intervention, but within the context of their professional organization or clinic rather than at the individual level. We collected data from both groups about current methods and best practices for dissemination, and the most appropriate channels and dissemination strategies to reach young adult adopters. The study was reviewed and approved by the University’s Institutional Review Board.

**Table 1:** Inclusion Criteria and Questions for Community Stakeholder Interview and Focus Group Participation

Focus group	Inclusion criteria	• Age 18–25 years • Not in a monogamous relationship (i.e., single, not dating; dating, not serious) • Have been in a monogamous relationship for less than 6 months and/or open to a sexual relationship with someone who is not their current monogamous partner • Had penile–vaginal sex in the past month • Have had an alcoholic drink at least once a week on average over the past 3 months • Have had one episode of heavy episodic drinking (4+/5+ drinks for women/men in one sitting) in the past month • Inconsistent use or nonuse of condom or contraception in the last month (women) • English speaker • Provides a valid phone number/email address and name • Willing to participate in focus groups; • Resides in Texas • Not pregnant or trying to get pregnant (women only) • Can provide valid government-issued photo ID to confirm identity
Community stakeholder interviews	Inclusion criteria	• Age 18 years or older • Employed at a healthcare clinic or community organization that serves young adults • English speaker • Provides a valid phone number/email address and name • Willing to participate in interview • Works in Texas

### Focus Groups

#### Recruitment

A convenience sample of Texas young adults, ages 18 to 25, were recruited through community organizations, universities, clinics, flyers, email invitations, social media sites, online postings, and on-site recruitment. Recruitment material contained a link to the study site and a brief screening survey to determine eligibility. The number of focus groups was predetermined based on similar studies among young adults (Lindgren et al., 2009; Thind et al., 2021). From the screening survey, contact information for those who were eligible and willing to participate in focus groups was collected. Focus groups were stratified by gender and were held via web-conferencing (e.g., Zoom). Before the focus groups, informed consent was confirmed verbally, and photo identification was verified to confirm age. A moderator and note-taker were present and sessions lasted about an hour. Participants were given a $50 gift card for their participation. Data collection occurred between February 2022 and April 2023.

#### Instrument

The focus groups used a semistructured focus group guide with additional probing questions. The research team developed draft content of the intervention based on previous content in the STARR intervention. Items were aligned with DOI constructs and included acceptability and feasibility of community-based intervention delivery (*intervention characteristics*) and preferred modes of accessing the intervention in real-world settings (e.g., community or clinical websites, advertisements; *dissemination and communication channels*). Table 2 highlights the sample questions for focus groups and interviews.

**Table 2:** Questions for Community Stakeholder Interview and Focus Group Participation

Focus group	Compatibility	• What do you think of this approach? ○ What did you like? Why? ○ What did you not like? Why? • What parts of the material do you think young adults would appreciate most or be interested by?
	Adaptability	• What would you change? • How often would you want to get a text message like this each week? • What time of day would text messages be the most useful? Least useful? • When do you think you be most likely to use these strategies? • How else could the timing and frequency of these messages be revised? • What could make the text messages more engaging and exciting? • How interactive would an ideal app or text messaging program be?
	Dissemination and communication channels	• How would you want to find out about a program like this? • How would you feel about getting text messages from a research study? • How would you feel about these messages being sent by an app installed on your phone rather than text messages?
Community stakeholder interviews	Relative advantage	• How does this program compare to other similar programs your organization uses? ○ What advantages does the intervention have compared to existing programs? ○ What disadvantages does the intervention have compared to existing programs?
	Compatibility	• How well does the program fit with existing work processes and practices in your organization? • Will the program replace or compliment a current program or process? ○ In what ways?
	Complexity	• How complicated is the program? ○ Consider: duration, scope, cost, number of steps involved, and whether the program reflects a clear departure from previous practices.
	Adaptability	• What kinds of changes or alterations do you think you will need to make to this program so it will work effectively for your clients ○ Do you think you will be able to make these changes? Why or why not?
	Dissemination and communication channels	• How would you notify your clients or potential clients about this program? ○ What information would you provide them with? ○ What strategies or channels would be best? • Would you notify the larger community about this program? ○ If so, how? • What role would your organization play in communicating about this program? • What additional ways could we communicate about this program to the target population?

### Stakeholder Interviews

#### Recruitment

A convenience sample of stakeholders were recruited from community organizations, universities, clinics, and other sites that serve young adults using flyers, social media, phone calls, and email invitations. Sample size was determined based on prior research (Hennink et al., 2019; Hennink & Kaiser, 2022). Participants emailed research staff or completed a brief online screening survey to determine eligibility for the study. Eligible participants were contacted via email to schedule an interview time, which was held via web-conferencing (e.g., Zoom, Table 1). Interviews were 30 minutes and all participants received a $50 gift card for their participation. Data collection occurred between April 2023 and May 2023.

#### Instrument

A semistructured interview guide was developed with DOI constructs but focused on acceptability of the modified community-based intervention (*intervention characteristics*) and future *dissemination and communication channels* to reach young adult adopters. Young adults and community stakeholders were also asked about ways to adapt the innovation to fit the needs of the intended audience (Table 2).

### Data Analysis

Focus groups and interviews were audio recorded and sent for transcription. De-identified transcripts were verified against the audio files and then entered into MaxQDA, a qualitative data analysis software. A codebook was developed based on *a priori* codes from the interview guide and constructs from the DOI described above. Emergent codes were added to the codebook as they were identified. Two researchers coded 10% of the transcripts for each method (two coders for focus groups, two coders for interviews), and reliability in coding was assessed (>90% for focus groups and >80% for interviews). One researcher coded the remaining transcripts for each method.

Once all codes were applied to the transcripts, themes, and relationships among codes were identified through axial coding (i.e., grouping codes into broader categories or themes based on discussion with the research team). Summaries of major themes were developed with descriptive reporting, and representative quotes were selected (Hennick et al., 2012). The coders from each method discussed and synthesized the data from the two data sources.

## Results

Demographic characteristics of community stakeholders (*n* = 12) and focus group participants (*n* = 10 focus groups; 16 young adults with 1–5 people per group; no overlap of participants in each group) are described in Table 3.

**Table 3:** Demographics of Focus Group Participants and Community Stakeholders

Characteristic	*N* (%)
Focus group participants	
Age	
18- to 20-year olds	2 (12.5)
21- to 25-year olds	14 (87.5)
Sex	
Female	5 (31.3)
Male	11 (68.8)
Race/ethnicity	
White non-Hispanic	5 (31.3)
Black or African American non-Hispanic	9 (56.3)
Asian non-Hispanic	1 (6.3)
American Indian/Alaska Native non-Hispanic	1 (6.3)
Community stakeholder interview participants	
Sex	
Female	9 (75.0)
Male	3 (25.0)
Race/ethnicity	
White non-Hispanic	6 (50.0)
Black or African American non-Hispanic	1 (8.3)
Hispanic	4 (33.3)
Other non-Hispanic	1 (8.3)
Setting	
Academic institution	6 (50.0)
Community organization/initiative	6 (50.0)

### Intervention Characteristics

#### Compatibility

Overall, there was acceptability of the proposed intervention by community stakeholders and focus group participants. Participants had a positive perception of the characteristics of the intervention as a whole. Participants described the need for young adults to learn about preventive strategies around sexual health, and some participants shared that the availability of the information would motivate them to act, as the messages serve as reminders. Some participants suggested this intervention may reach a population that might not be reached otherwise and reach individuals who are ready to change their behaviors.

I wouldn’t mind incorporating that into some of the program offerings that we have. It kind of checks all the boxes as far as programming goes. (P12, Stakeholder, Man)

I would like to try it out, but the fact that these things are available might prompt me to just give it a try and that’s it. This would definitely make me want to act on the information I just got. (FG1, Young Adult, Man)

Participants also discussed that text messages may be an effective strategy in reaching young adults, especially when compared to apps that must be installed. The text messages were seen as a strategy to encourage safe sex and responsible behavior during alcohol use, thereby protecting individuals and their partners. Participants described that text-based interventions are readily accessible to the intended population of young adults, given their current behaviors related to communication via text.

I see it as unique approach . . . it’s going to be an effective one because talking about text messaging, this is because young adults my age, we are mostly on our devices 24 hours a day. If there’s an effective way to communicate to us, it’s definitely through mobile devices. Using text messaging seems to be quite an effective means to achieve that goal. (FG1, Young Adult, Man)

I really think that the text messaging is just simple and very efficient and doesn’t want to put much strain on installing and update apps on the phone. (FG4, Young Adult, Man)

I think having the texts like they do, they’re always on their phones, having some sort of intervention through their technology or through social media platform or something could work. I think text is a good modality. (P2, Stakeholder, Woman)

#### Adaptability

Participants provided suggestions on ways to ensure that the intervention and message content can be tailored to the intended audience. There was a strong emphasis on customization and adaptability, not only in timing but also in content, ensuring messages are relevant to the recipient’s lifestyle and sexual practices. Participants described that messages should be engaging and relevant to young adults. Participants also shared the need to consider personalizing the topics and content related to behavior change. This includes consideration of gender-inclusive language and the specific needs of diverse groups, such as queer individuals, to avoid further stigmatization and ensure the effectiveness of the messages.

Making sure that the messages apply to the individual receiving them is important and also taking care to apply the appropriate gendered language. (FG10, Young Adult, Man)

Maybe certain general topics or areas that they’re interested in wanting to change or learn more that they might receive more of those types of texts, rather than the ones that aren’t as helpful. (P2, Stakeholder, Woman)

#### Design Quality and Packaging of Intervention and Content

Concerns about the frequency of intervention messages were discussed by some participants, highlighting that some individuals may find it overwhelming. This could discourage individuals from continued participation. Most participants suggested a frequency of two to three times per week as sufficient to keep the information relevant and top-of-mind without becoming overwhelming or intrusive.

The con to that is really what I was talking about earlier. You may get to a point to where you’re like, “I’m overwhelmed and I have all of these text messages to go through and maybe I just don’t answer that one.” (P7, Stakeholder, Woman)

Maybe two actually. The initial one and maybe one later in when the evening itself, maybe now when someone is actually drinking or actually might be the possibility of actually having sex. (FG7, Young Adult, Man)

Finally, participants expressed a consensus that reminders about sexual health should prioritize information on STIs and prevention more than pregnancy prevention. They argue that while many people feel they have control over pregnancy, STIs represent a less-controllable risk, making awareness of them more critical. Participants also suggested that current statistics on sexual activity and alcohol use among young adults should be provided as additional references, particularly acknowledging social norms.

When it comes to combating the perception that everybody’s drinking a lot, everybody’s hooking up, these text messages could challenge that. Maybe it’s a random fact, “Did you know that only X person of people have sex? Did you know that 97% of your peers report using condoms when they. . .?” Like having some norms or social norms presented, but then maybe even questions like, “What’s your plan for tonight?” It could have them pause and kind of think about that ahead of time and set that plan while they’re sober. (P2, Stakeholder, Woman)

The least useful reminder should be about pregnancy because. . .the world is getting smarter and everyone has this control over child bit and all this stuff. People are smarter these days, but I think the best part of this reminder should be really focused on the STI and the risks, because no matter how smart you are, you are not that smarter than the infection. So instead of hammering on pregnancy, which people ignore and feel they gain control over it, you should remind them of what they can’t control, which is the infection. (FG4, Young Adult, Woman)

### Dissemination and Communication Channels

#### Social Media

For strategic ways to communicate and disseminate the proposed intervention, young adult and stakeholder participants mentioned social media (Instagram, Twitter, Snapchat, TikTok) in reaching most young adults. Some individuals discussed specific methods to use in communication, such as reels or videos showing how the intervention could be used, to increase awareness and adoption of the intervention.

TikTok, Instagram, the very big ones right now. You could try to target Facebook, but I feel like it’s mostly older generation that’s on Facebook now. (FG8, Young Adult, Woman)

Social media plays a big role too. With Instagram, Twitter, Snapchat, those types of platforms. I just know that our students are on them constantly. Not really Facebook as much, but Instagram and TikTok. Showing a reel on how this works could maybe help influence their decision to say, “Well, I’m excited for that.” (P2, Stakeholder, Woman)

Young adult participants preferred receiving communication about the intervention via text messages rather than apps. Participants shared that text messages were easily accessible and can get the attention of the intended audience and refer out to a webpage or site with other information to introduce the intervention.

A text message coming in first might be like giving the central idea of the program, then can come with a link. If you click, it will take you to a webpage for a full description. Whoever receives the message can have it at the back of the mind to click a link in the message, that’s a free time to learn more about it. The text message that comes first shouldn’t be intruding, something simple just with the central idea of the study. (FG1, Young Adult, Man)

#### Events and Campus Resources

Participants from both groups shared that social events within campuses are a great avenue to increase awareness. Participants shared that university and school settings, such as through student organizations and classrooms, were also ways to disseminate information about the intervention. Community resource fairs were also highlighted as potential venues. Other methods, such as flyers and resource lists, may be useful in increasing awareness of the resources available and encourage individuals to reach out at their own time. Quick response codes (QR codes) were also mentioned as a convenient and quick way for individuals to get information and should be included in the information materials.

It would be beneficial at places like resource fairs and pride and other places that are typical for people who are actively looking for resources or ways to contribute to the community and the community knowledge base. (FG10, Young Adult, Man)

Also looking at different events that might be taking place. If there’s a big event that’s happening in the summer, maybe it’s a career fair, maybe it’s a wellness event or expo, maybe sponsoring something. One of the benefits of sponsoring could be them publicizing what you’re doing or having a table there where you can have those flyers with the QR codes. (P7, Stakeholder, Woman)

### Discussion

Given the efficacious findings of a previous web-based intervention designed to reduce alcohol-related sexual health outcomes (Lewis et al., 2019), expanding the intervention to be text-based and include contraception-related considerations can potentially reduce negative outcomes in population disproportionately at risk for unwanted pregnancies (CDC, 2024; Guttmacher Institute, 2019). This qualitative examination of DOI constructs among a sample of focus group participants and key community stakeholders identified several themes related to intervention compatibility, adaptability, and acceptability of messaging and delivery, along with strategies to increase intervention dissemination. Findings support young adults’ willingness to receive tailored text messages and opportunities for dissemination through several community organizations.

These results underscore participants’ acceptability of the proposed intervention, particularly the text message-based approach. Intervention acceptability suggests that despite declining rates of sexual activity among young adults (Kaiser Family Foundation, 2023; Lei & South, 2021), there may be interest in conversations around sexual health that can lead to positive promotion opportunities. Moreover, participants indicated a need for information about STIs rather than pregnancy prevention, suggesting pregnancy information may be more widely available than STI prevention content. This desire for more information on STIs may be linked with most U.S. states requiring sexual health education to emphasize abstinence (SIECUS, 2022), which continues to be a politically contentious issue. Examining the associated equity issues in accessing information related to STIs may be an important next step in research as significant disparities exist in STI diagnoses for young adults but also among sexual and gender minority individuals and racial and ethnic minority groups (CDC, 2021). This research may provide guidance about the educational context in which this intervention’s information will be delivered to allow for further adaptation to meet the needs of the end users.

The dissemination and communication channels identified were similar between community stakeholders serving young adults and focus group participants. Both groups identified social media apps as a method to share information quickly and increase overall awareness and engagement with the intervention. Moving forward with social media as a dissemination channel, future research should develop strategies informed by evidence-based best practices to be effective (Stellefson et al., 2020). In addition to selecting appropriate channels, determining the timing and volume of posts, measuring engagement and interaction (Schillinger et al., 2020; Stellefson et al., 2020), and developing a comprehensive intervention communication plan using a dissemination science framework can increase awareness and adoption of the intervention and prepare for wider scale-up (Baumann et al., 2022).

The overall acceptability of a text-based intervention approach is noteworthy, given the potentially fleeting nature of social media app popularity. For example, from the time of conducting this study to publication, the social media app Twitter (now X) underwent substantial changes, including in name, ownership, and regulation, all of which may impact audience reach (Tenore, 2023). Similarly, the U.S. federal government continues to explore potential restrictions and bans on some social media apps, such as TikTok, due to national security concerns (The Federal Register, 2023). Preferences for text messaging, in addition to changing app dynamics and possible federal intervention on app usage, may suggest compelling reasons for favoring text message–based interventions over apps and provide evidence to explore additional dissemination channels for future communication.

### Strengths and Limitations

The goal of this study was to inform the adaptation of an efficacious intervention to both understand how to disseminate to young adults and how to incorporate information regarding contraceptive use. The dual use of effective contraceptive methods and condoms is ideal for the prevention of STIs and unintended pregnancies for pregnant-capable persons. However, during this 2-year period, we had difficulty in identifying participants who met the inclusion criteria of both nonuse or inconsistent use of contraception and condom nonuse, while also engaging in heavy episodic drinking. Based on our screening data (not shown), the high-risk inclusion criteria made it challenging to identify eligible participants despite multiple recruitment modalities. Thus, we did not meet our recruitment goals for women in this study. Given that this was a pilot study to solicit feedback and assess the feasibility of recruiting this specific population for this adapted intervention, this points to a potential lack of engagement among young adults at present (National Center for Complementary and Integrative Health, 2023). Next steps for this study may expand to include young adults with moderate or low-risk behaviors to prevent more high-risk sexual and alcohol-related behaviors, rather than limiting the sample by including only those with the current behaviors.

These findings may be limited in transferability, given the restrictive eligibility criteria as described above. For example, results about STIs being more important than pregnancy prevention may be skewed because there was a less-representative sample of pregnancy-capable individuals. Since focus groups required video conferencing technology and participants were required to show photo identification, the sample may not be representative of populations with less technological resources or those who do not have photo identification, who may be less accepting of a text message–based or mobile phone–related intervention. Other potential selection bias may be present (e.g., people with a favorable view of text-based interventions may be more likely to participate, thus eliciting more positive feedback), but several procedural measures were taken to notify participants of the protections made to allow them to speak openly and honestly. In addition, the absence of specific population groups highlights the potential disparities in sexual and reproductive health outcomes, as those with reduced access to technology or without photo identification may already face greater inequities. Addressing these disparities in the next steps of this line of research is crucial to ensure more equitable health interventions and outcomes.

### Implications for Practice

The results from this study, particularly the dissemination and communication channels identified, have implications for public health practice and health education. For example, in addition to social media, specific channels of communication were mentioned, including flyers, college settings, social events, and community resource fairs, all of which are commonly reported as appropriate channels for health programming by young adults (Dobbs et al., 2020; Koskan et al., 2022; Wordlaw & Vilme, 2024). This information may be beneficial to health educators working with this population, particularly when developing or adapting other interventions related to sexual health or alcohol prevention. Moreover, the more immediate finding from this pilot study is the identification of the channels for disseminating this efficacious web-based intervention to reach a broader audience of young adults. Moving forward, we suggest partnering with these types of organizations or channels interested in using this intervention to address community needs related to alcohol and sexual health for young adults.

### Implications for Research

Involving the end users and stakeholders in the development of the intervention, as seen in this study with both intervention design/packaging and dissemination strategy development, can result in improvement in adoption and sustainment in the future (Kwan et al., 2022). Researchers focusing on intervention adaptation or development should consider *designing for dissemination and sustainability (D4DS)*, an approach that promotes beginning intervention development with the end user in mind, considering the fit within the context, and examining the future implementation and sustainability of the intervention (Kwan et al., 2022). Using a D4DS approach could be explored further in studies addressing intervention development and dissemination. Integrating the science of dissemination and implementation into intervention planning and development is essential, so research can more rapidly translate into clinical practice. Moreover, novel strategies are needed to recruit the young adult population who are outside of the college setting and within a broader community setting. This can assist with supporting the transferability of research findings.

## Conclusion

This theory-informed qualitative study serves as a preliminary step to adapting an efficacious personalized intervention and determining appropriate channels for communication. Feedback from young adults and community stakeholders highlighted key considerations regarding intervention characteristics and dissemination. Findings of this study can inform the continued adaptation and implementation of the STARR intervention, with next steps including assessing the effectiveness and efficacy of the intervention on improving sexual health outcomes. This pilot study will also focus on expansion to recruit a larger, more diverse sample of young adults and continue to identify the unique, community-derived factors that may be addressed to help reduce negative outcomes related to condomless sex and unintended pregnancy in young adults.
